# Evaluating adults’ health-related values and preferences about unprocessed red meat and processed meat consumption: protocol for a cross-sectional mixed-methods study

**DOI:** 10.12688/f1000research.23593.2

**Published:** 2021-05-13

**Authors:** Claudia Valli, Victoria Howatt, Anna Prokop-Dorner, Montserrat Rabassa, Bradley C. Johnston, Joanna Zajac, Mi Ah Han, Fernando Kenji Nampo, Gordon H. Guyatt, Malgorzata M. Bala, Pablo Alonso-Coello

**Affiliations:** 1 Department of Paediatrics, Obstetrics, Gynaecology and Preventive Medicine, Universidad Autónoma de Barcelona, Barcelona, Spain; 2 Iberoamerican Cochrane Centre, Biomedical Research Institute San Pau (IIB Sant Pau), Barcelona, Spain; 3 Faculty of Medicine, Dalhousie University, Halifax, Nova Scotia, Canada; 4 Department of Medical Sociology, Chair of Epidemiology and Preventive Medicine, Jagiellonian University Medical College, Krakow, Poland; 5 Department of Health Research Methods, Evidence and Impact, McMaster University, Hamilton, Ontario, Canada; 6 Department of Community Health and Epidemiology, Dalhousie University, Halifax, Nova Scotia, Canada; 7 Department of Nutrition, Texas A&M University, College Station, Texas, USA; 8 Department of Hygiene and Dietetics, Chair of Epidemiology and Preventive Medicine, Jagiellonian University Medical College, Krakow, Poland; 9 Department of Preventive Medicine, College of Medicine, Chosun University, Gwangju, South Korea; 10 Latin-American Institute of Life and Nature Sciences, Federal University of Latin-American Integration, Evidence-Based Public Health Research Group, Foz do Iguassu, Brazil; 11 Department of Medicine, McMaster University, Hamilton, Ontario, Canada; 12 Chair of Epidemiology and Preventive Medicine, Department of Hygiene and Dietetics, Jagiellonian University Medical College, Krakow, Poland; 13 CIBER de Epidemiología y Salud Pública, (CIBERESP), Barcelona, Spain

**Keywords:** health, values and preferences, red meat, processed meat, cross-sectional study, mixed methods

## Abstract

**Background: **People need to choose from a wide range of foods, and in addition to availability and accessibility, people’s values and preferences largely determine their daily food choices. Given the potential adverse health consequences of red and processed meat and the limited knowledge on individuals’ health-related values and preferences on the topic, such data would be useful in the development of recommendations regarding meat consumption.

**Methods and analysis:** We will perform a cross-sectional mixed methods study. The study population will consist of adult omnivores currently consuming a minimum of three weekly servings of either unprocessed red meat or processed meat. We will explore participants’ willingness to stop or reduce their unprocessed red meat, or their processed meat consumption through a direct-choice exercise. This exercise will consist of presenting a scenario tailored to each individual’s average weekly consumption. That is, based on a systematic review and meta-analysis of the best estimate of the risk reduction in overall cancer incidence and cancer mortality, we will ask participants if they would stop their consumption, and/or reduce their average consumption. We will also present the corresponding certainty of the evidence for the potential risk reductions. Finally, we will measure their meat consumption three months after the interview and determine if they have made any changes to their average consumption.

**Ethics and dissemination:** The research protocol was approved by the ethics committees in Canada (Research Ethics Board, Dalhousie University), Spain (Comitè Ètic d'Investigació Clínica de l'IDIAP Jordi Gol), Poland (The Bioethics Committee of the Jagiellonian University), and Brazil (National Research Ethics Commission). The study is based on voluntary participation and informed written consent. Results from this project will be disseminated through publications and presentations.

## Introduction

Food choices are important for the overall health of each individual ^1^. On a daily basis, people need to choose from a wide range of food in order to meet their nutritional requirements ^2^. People’s dietary values and preferences influence the types of foods they consume, as well as the quantity of consumption ^3, 4^. However, nutritional guidelines have consistently ignored the systematic identification and incorporation of people’s values and preferences in the development of their recommendations ^5, 6^.

In light of recent studies showing an association between unprocessed red meat and processed meat consumption and adverse health outcomes, such as all-cause mortality, cardiovascular mortality, cancer risk, and stroke ^7– 12^, dietary guidelines have generally endorsed limiting meat intake (e.g. limiting processed meat) ^13– 15^. However, limited information exists regarding how much people value meat in their diet and their willingness to reduce meat consumption in the face of undesirable health effects ^16^. Recently, an international panel of 14 members noted the low quality evidence supporting the causal relation of meat and adverse effects, and the small protective effect of reducing meat consumption if indeed such an effect exists. The panel formulated a weak recommendation in favor of continuing usual consumption ^17^. The recommendation was also based on a systematic review of studies addressing peoples’ values and preferences regarding meat consumption; however, the evidence was also judged to be of low quality given identified issues with risk of bias and indirectness ^16^.

We have therefore designed a study to evaluate adults’ values and preferences regarding meat intake and their willingness to change their consumption in the face of possible undesirable health consequences. Given the general importance of reducing cancer, the recent claims on cancer risk associated with meat consumption from the International Agency for Research in Cancer and the World Cancer Research Fund ^8, 18^, and in an attempt to avoid overwhelming participants with too much information, based on a systematic review of the literature ^11^, we chose the risk estimates for two cancer outcomes to share with participants, specifically cancer incidence and cancer mortality.

This study is part of NutriRECS (Nutritional Recommendations; www.nutrirecs.com) ^19^, an initiative that aims to: 1) apply rigorous systematic review and guideline methods using the GRADE approach to investigate the association between diets, foods and nutrients and health outcomes; 2) incorporate patient and community values and preferences to inform guideline recommendations; 3) apply strict and transparent management of conflicts of interest, and; 4) disseminate nutritional recommendations via open-access peer-reviewed publication.

## Methods and analysis

### Study design and setting

We are conducting an international cross-sectional mixed-methods study including: i) a quantitative assessment through an online survey; ii) followed by a qualitative evaluation through semi-structured interviews and, iii) a follow-up quantitative assessment through a questionnaire in four three different sites in four three countries (Spain, Brazil, Canada and Poland). Study settings will include primary health care centers, universities, and the general community. The study began in 2019 with recruitment and data cleaning ongoing, with expected completion in early 2021. In 2019, we conducted a pilot study in a sample of 32 participants recruited in the general community in Nova Scotia and Prince Edward Island, Canada (20). The results and feedback of this pilot study were used to inform and improve the study’s procedures.

### Study population and eligibility criteria

We will enroll adults 18 to 80 years of age who currently consume a minimum of three serving per week of either unprocessed red meat or processed meat. Unprocessed red meat is defined as mammalian meat (e.g. beef, pork, lamb), and processed meat is defined as white or red meat preserved by smoking, curing, salting, or by the addition of preservatives (e.g., hot dogs, charcuterie, sausage, ham, and cold cut deli meats) ^21^. We will exclude adults who have active cancer; those who have severe cardiovascular disease (history of stroke, acute coronary syndrome, heart failure, and symptomatic peripheral arterial disease); those who are pregnant; and participants unwilling or unable to provide informed consent.

### Recruitment strategy

We will recruit convenience samples of participants from the general population or people studying or working at universities. We will recruit participants from the general population using social media postings on the Cochrane website, Twitter, and Facebook pages. We will recruit people studying or working at universities by email. The social media posting and the emails will include information on study’s details, eligibility criteria, contact information of the researcher carrying out the study, and the related link to access the online survey.

### Sample size

For the quantitative assessment, we have made a best estimate of the proportion willing to reduce their meat intake of approximately 0.5 using the response distribution results from our pilot study based on a proportion of 0.53 of pilot participants willing to reduce unprocessed red meat and 0.44 of participants willing to reduce processed meat ^20^.We decided that a margin error around this estimate of as ± 0.1% is acceptable. We can achieve this precision with a 0.5 estimate in our primary outcome, the proportion of individuals ready to reduce or stop eating meat. Our sample size estimate is 96 participants at each site (95% confidence interval with ± 0.1% margin error) ^22, 23^.

For the qualitative evaluation, through a maximum variation sampling strategy, in each site, we will include participants until data saturation. Data saturation is achieved when no additional concepts emerge ^24^. During data collection and analysis, if the research team determines that we have not reached data saturation, recruitment will be extended to include more participants until saturation is achieved. The maximum variation technique consists of the inclusion of a highly heterogeneous sample, and a description of the variability or dispersion for the relevant variables ^3, 25^. We will attempt to include an approximately equal number of participants with the following characteristics of these variables: gender (men and women); age (those between 18 to 66 years old, and those between 67 and 80 years older); education level (those with some high school or less, those with a high school degree, and those with a college degree) and willingness to stop or reduce meat consumption (willing ≥5 from the Likert-Scale and unwilling ≤4 from the Likert-Scale).

### Study procedures

For the quantitative assessment, participants interested in participating will access the online survey and will be able to complete the questionnaire, including demographic characteristics, medical history information and meat consumption beliefs and behavior. The questionnaire will also include a direct choice exercise that will consist in presenting scenarios tailored to each individual’s typical weekly meat consumption. These scenarios will reflect the best estimate of absolute risk reduction in overall cancer incidence and cancer mortality over their lifetime based on our systematic review and dose-response meta-analysis ^11^. This will allow us to assess participants’ willingness to: a) stop or b) reduce their unprocessed red meat and processed meat intake in the face of overall cancer incidence and cancer mortality risks.

After presenting participants with the cancer incidence scenario tailored to their consumption, participants will be asked regarding their willingness to stop their unprocessed red meat intake. If participants will be unwilling to stop (≤4 of the Likert-scale), they will be presented with an additional question about their willingness to reduce. Similarly, participants will be then presented with the cancer mortality scenario and related questions for unprocessed red meat. Finally, participants will be presented with the cancer incidence and mortality scenarios tailored to their processed meat consumption with the same logic of questions explained above. 

Participants will be presented with both scenarios of unprocessed red meat and processed red meat. If participants declare to consume less than one serving of one type of meat per week, for example unprocessed red meat, they will skip the questions on red meat and will be presented with the scenarios and questions of processed meat only and vice versa. Finally, we will conduct a follow-up assessment, either by phone or by email, at three months to ask participants, who agreed to be contacted, if they have made any changes in their meat consumption.

***Questionnaire.*** Based on our pilot study, we further developed and piloted a questionnaire in each site to collect the following data: age, sex, socioeconomic status, educational level, employment status, household size, religious beliefs, the presence of chronic and other health conditions, and family history of cancer, and meat consumption beliefs and behavior information. We asked both men and women with different educational backgrounds and of different ages (those between 18 to 66 years old, and those between 67 to 80 years older) to complete the questionnaire in order to identify ways of improving the content and/or structure of the questionnaire.

We will assess participants’ current weekly consumption of unprocessed red meat and processed meat. We will facilitate these questions related to their meat consumption habits by providing pictures illustrating types of meats and serving size to determine the typical number of servings they consume of each meat weekly. In addition, we will determine which factors participants take into account when choosing their diet, whether their food choices influence or are influenced by other people (e.g. preparing food for children) and to what extent they are satisfied with their current diet. See *Extended* data for the Spanish version of the online survey.

***Serving size estimate and participant’s current meat consumption assessment.*** We estimated that each serving of unprocessed red meat is equal to 120g, and 50g for processed meat ^11^. In Spain, the mean ± standard deviation of meat intake, according to 2016 Spanish National dietary survey in adults, conducted by the Spanish Agency for Consumption, Food Safety and Nutrition, is 37 ± 63g/day (2 servings/week) of unprocessed red meat and 32 ± 65g/day (4 servings/week) of processed meat ^26^. In Brazil, according to the Health Survey conducted in São Paulo in 2008, the mean ± standard error of meat intake is 71 ± 2 g/day (4 servings/week) of unprocessed red meat and 28 ± 1 g/day (4 servings/week) of processed meat ^27^. In Poland, according to the domestic deliveries and consumption report of 2017, the average intake of both unprocessed meat and processed meat is 115 g/day (9 servings/week) ^28^. In Canada, according to the Statistics Canada’s Canadian Community Health Survey, the mean intake among Canadians is 52 g/day (3 servings/week) of unprocessed red meat and 22 g/day (3 servings/week) of processed red meat ^29^. Based on these data, we defined the average intake of both unprocessed red meat and processed meat as 3 servings per weekto calculate the baseline risks of cancer incidence and cancer mortality. In order to assess participant’ current meat consumption, we determined the absolute risk reduction for all meat consumption frequency categories (servings/week) as follows: 3 to 4, 5 to 6, 7 to 8, 9 to 10, 11 to 12, 13 to 14, and more than 15 servings per week. We will report in servings per week their current meat consumption for both unprocessed red meat and processed meat.

**Figure 1.**
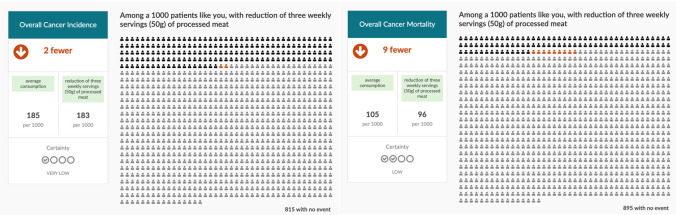
Three servings/week scenarios for processed meat.

**Figure 2.**
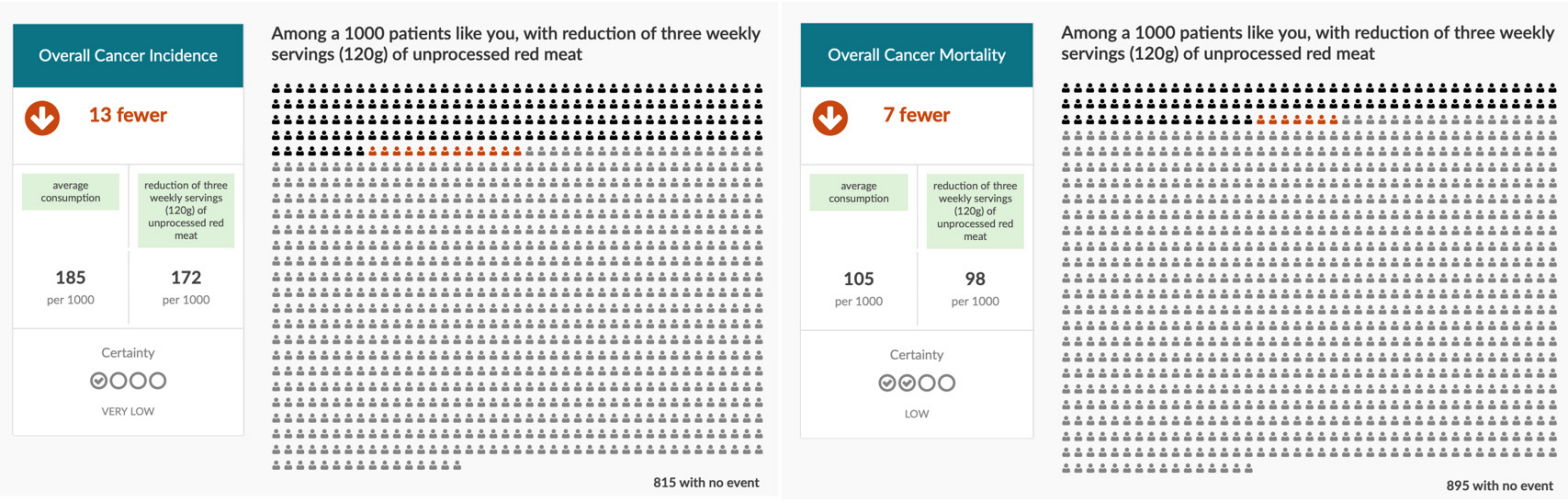
Three servings/week scenarios for unprocessed red meat.

***Direct choice exercise.*** Following standard methodologies used in previous work in the field of obstetrics from members of our team ^30, 31^, we will use a direct choice experimental design to assess the proportion of people willing to change their consumption when faced with a risk reduction of overall cancer incidence and cancer mortality based on a seven point Likert- scale from 1 (meaning definitely not) to 7 (meaning definitely yes). To ensure that participants have a similar understanding of these two outcomes, we will describe the development of each outcome through the use of health states examples ( Table 1 and Table 2). We will present our data from our systematic review that addressed the possible impact of reducing meat intake on overall cancer incidence and mortality ^11^. We will first present the baseline risk and the risk reduction participants might achieve by stop eating meat and its certainty. We will develop an interactive electronic decision aid using MagicApp software ( http://magicproject.org/research-projects/share-it/) to show the probabilities of reducing the risk of overall cancer incidence and overall cancer mortality if participants’ would stop eating unprocessed red or processed meat (three servings/week scenarios in Figure 1 for processed meat and Figure 2 for unprocessed red meat intake – see *Extended data* ^32^ for all servings/week scenarios ). In addition to the risk reductions, the overall certainty of evidence based on the GRADE approach for cancer incidence and mortality will be shared with the participant ^33^. For the direct choice exercise in the online survey, we will provide an explanatory video that will describe to participants how to read and interpret the data presented in the scenarios. In addition, we will provide participants with explicit text tailored to their average weekly meat consumption. If participants are unwilling to stop eating meat to achieve the possible associated health benefits, we will ask them if they would be willing to reduce their meat intake but remind them that the cancer risk reduction, they might anticipate will be less by reducing their meat intake then stopping completely.

**Table 1.** Health states - Cancer incidence.

Cancer incidence	
**Symptoms & Signs**	• Cancer is wide group of diseases and may cause many signs or symptoms • Some signs and symptoms are common for different cancers while others are more specific for each type of cancer • Not explained loss in body weight, night sweats, fever • Problems with eating, loss of appetite • Weakness/ fatigue • Sometimes bleeding or discharge, blood in stool or urine • Change in bowel habits, difficult or painful urination • Pain • Unexplained anemia • Persistent cough or blood in saliva • Persistent lumps or swollen glands • Changes on the skin
**Treatment**	• There are different types of treatment that will depend on the type of cancer and how the cancer is advanced. • You may receive only one treatment, but in most cases a combination of subsequent is needed: surgery and/or hormone therapy (giving hormones or drugs that block hormones to slow down cancer growth), chemo or immunotherapy (drugs that kill cancer cells or flag them for immune system to destroy) and/or radiation therapy (radiation in high doses to kill cancer cells or slow their growth).
**Consequences**	• You can experience side effects of cancer treatment, such as anemia, loss of appetite, fatigue, hair loss, nausea • You can experience pain, gastrointestinal problems, urinary problems • It will affect your social life short term and possibly long term • You can experience long-term consequences of cancer and its treatment, such as problems with heart, lungs, endocrine system, bones and joints, digestion, memory • You may experience anxiety, depression and other emotional problems • You may no longer be able to participate in your regular activities • You may die

**Table 2.** Health states – Cancer mortality.

Cancer mortality	
**Symptoms & Signs**	• Before you die you experience symptoms related to cancer and its spread, such as pain, weakness/fatigue; those symptoms may have various duration, you may suffer those symptoms for several years • Before you die you experience unwanted effects of treatment you received for cancer. • You are dead and you do not feel any pain or breathlessness.
**Treatment**	• There is no need for any treatments and they are stopped
**Consequences**	• You lose your vital bodily and mental functions, ending your life. • You will leave everything that was important in short time span.

***Semi-structured interview.*** We will also develop and pilot a script in each site for a semi-structured interview. We will conduct these interviews in order to explore peoples’ motives regarding their willingness to change their meat consumption. Based on our pilot study, interviews will take approximately 30 minutes. See *Extended data* for the Semi-structured interview script.

***Follow-up assessment.*** We will contact participants by phone or by email three months after the online survey and ask them if they have made any changes in their meat consumption. In case of the phone follow-up, we will follow a semi-structured telephone script previously piloted; in instances where participants prefer to be contacted by email, we will send them a questionnaire with the same content we will use for the phone interview. See *Extended data* for the Follow-up assessment script.

### Outcomes

The primary outcome measure for all included participants will be willingness to change meat consumption in the face of the undesirable cancer health risks. We will show participants the cancer risk reduction they may achieve if they would stop eating unprocessed red meat or processed meat tailored to their weekly consumption and ask them if they are willing to stop, on a scale from 1 (meaning “definitely not”) to 7 (meaning “definitely yes”). If participants are not willing to stop eating meat (≤4 from the Likert-scale), we will ask them if they will be willing to reduce any amount of their weekly meat intake, on a scale from 1 (meaning definitely not) to 7 (meaning definitely yes). As a secondary outcome, we will explore participants’ values and preferences regarding meat intake and the related motives around their willingness or unwillingness to make any changes. We will ask participants in the qualitative evaluation, which factors determine their unprocessed red meat or processed meat intake, and to what extent these factors influence their willingness/unwillingness to stop/reduce their meat consumption. Finally, we will estimate their meat consumption at three months after the online survey and determine if they have made any changes.

### Data synthesis and analysis

***Quantitative analysis.*** We will describe participants’ demographic and medical history information as well as meat consumption behaviors using means and standard deviations or frequencies and proportions, as appropriate.

We will describe the distribution of the continuous dependent variables: a) “willingness to stop unprocessed red meat consumption in the face of cancer incidence risk”; b) “willingness to stop unprocessed red meat consumption in the face of cancer mortality risk”; c) “willingness to reduce unprocessed red meat consumption in the face of cancer incidence risk”; d) “willingness to reduce unprocessed red meat consumption in the face of cancer mortality risk”, by presenting histograms and using means and standard deviations or median and IQR, as appropriate. We will do the same analysis for processed meat. Then, we will conduct an exploratory linear regression analysis using the above dependent variables and the participants’ characteristics (sex, age, level of education, occupational status, religious belief, and family history of cancer) as the independent variables. We will calculate the beta coefficients and the associated 95% confidence interval of participants who are willing to avoid, and for those willing to reduce unprocessed red meat and processed meat consumption in the face of undesirable cancer risks.

Additionally, we will conduct an exploratory logistic regression analysis using the dependent variables on willingness as categorical variables: those willing (≥5 from the Likert-Scale) and unwilling (≤4 from the Likert-Scale). We will calculate the odds ratio and the associated 95% confidence interval of participants who are willing to avoid and reduce meat consumption in the face of undesirable cancer risks.

Using our three-month follow-up assessment data, we will calculate the frequency and proportion of participants who made any changes in their meat consumption.

***Qualitative analysis.*** We will audio-record and transcribe verbatim all semi-structured interviews and use thematic analysis for the qualitative analysis ^34, 35^. For our iterative analysis, we will use constant comparison within and across cases to identify any patterns. We will code all transcripts and then the codes will be sorted into themes. We will subsequently compare the identified themes with demographic and participant characteristic information collected to demonstrate any patterns among groups such as sex, age, and education level.

***Integrating qualitative and quantitative analyses.*** We will conduct a sequential analysis of the quantitative and qualitative components of the data. We will analyze each dataset separately and then, at the end of the study, draw meta-inferences informed by the findings from both data sets. We expect the qualitative results to provide a better understanding of the decision-making process than if the quantitative results were considered alone.

### Ethics and dissemination

Research approval was obtained by the Research Ethics Board, Dalhousie University (Canada; 2019-4715), the Clinical Research Ethics Committee of the Jordi Gol University Institute for Primary Care Research (IDIAP; Spain; 19/121-P), the Bioethics Committee of the Jagiellonian University (Poland; 1072.6120.141.2019), and the National Research Ethics Commission (Brazil; CAAE 21826419.4.0000.8527), and if needed will be obtained from all other participating sites. We will explain the entire process of the study to the participants and we will present the potential benefits and risks of participation. The potential benefits of this study to participants include gaining an understanding of the current research regarding overall cancer mortality and incidence based on an up to date high quality dose-response systematic review and meta-analysis ^11^, which participants could use in future dietary decisions. There are no potential physical or psychological risks to participating in this study.

Participation in the study is voluntary and participants may withdraw from the study at any time without penalty. Should they choose to withdraw; participants will decide whether they want us to discard all or some of the data they have provided. Participants willing to participate will have to sign a written consent form, and they will be assigned a number to anonymize all data collected. Consent forms will be kept separately in a secure cabinet. All interviews will be audio-recorded and transcribed onto a computer file. The recording device will be stored in a secure cabinet and the recordings will be deleted upon completion of the study. Participants will not be identified by name nor otherwise identified when research results are shared. It is possible that a participant could be quoted to highlight results, however, they will be anonymized and neither their name, nor their assigned alphanumeric code, will be shared. Participants will be made aware of this possibility during the consent process and may, if they wish, choose not to allow the use of direct quotations. No compensation will be provided to participants. We will share with participants a copy of our published final results by email or by postal service.

We will adhere to the checklist of good practice in the conduct and reporting of survey research ^36^ when reporting our results. Results will be disseminated through publications and presentations.

## Discussion

Our international mixed-methods study will be the first to explicitly explore peoples’ health-related values and preferences, and their willingness to stop and/or reduce meat consumption when informed of the potential adverse cancer risk, and the uncertainty around this evidence. The information patients will receive will be based on a recent systematic review and dose-response meta-analysis ^11^.

### Our study in the context of previous research

Because there is limited information in the literature on how people value their health in relation to their diet, developing nutritional recommendations based on health-related values and preferences of community members is a major challenge. Previous studies addressing people’s meat preferences did not adequately present the undesirable health effects of meat consumption in ways that captured the current evidence and its uncertainty ^37, 38^.

In the context of the NutriRECS initiative, our team conducted a systematic review that summarized evidence that omnivores are attached to meat and are reluctant to reduce their meat consumption. However, we rated the certainty of evidence as low due to issues with risk of bias, indirectness, and because of the small number of participants and limited information regarding data analysis ^16^.

A NutriRECS international panel using an individual patient perspective formulated a weak recommendation in favor of continuing current unprocessed red meat and processed meat consumption, acknowledging the low certainty regarding the values and preferences evidence ^17^. This experience triggered the design of the present study, aiming to overcome the limitations of the studies to date ^16^.

### Limitations and strengths

Our study has some potential limitations. Our sample includes participants living in high-income countries or from high income strata in low to middle income countries. Therefore, we cannot generalize these findings to low-income populations. We will, however, collect information on participants’ socioeconomic status and education level in order to explore the effect of these characteristics on participants’ dietary values and preferences.

A second limitation of our study is the exclusive focus on cancer outcomes, despite evidence suggesting that reducing meat consumption may reduce the risk of diabetes and cardiovascular outcomes ^12, 39^. However, due to the recent claims of meat consumption and cancer risks ^8, 40^, the inconsistency in data on cardiometabolic risk associated with both unprocessed and processed meat ^10, 39^, and to not overburden participants with too much information, we prioritized two cancer outcomes.

Regarding strengths of our study design, we will address some of the limitations in the previous studies by following a systematic and transparent approach with the use of questionnaires, direct choice exercises and open-ended questions to assess peoples’ health values in relation to their unprocessed red meat and processed meat consumption. We will inform people of the most recent evidence of meat consumption and its related cancer risks ^11^, including the certainty of evidence for these risks, according to their current weekly average consumption. In addition, we will explore their willingness to make any changes to their diet based on the potential risk reduction in cancer.

Our international multicentre study will help ensure generalizability of the results. In addition, the collection of both quantitative and qualitative data will enable an accurate identification of the current health values and preferences regarding meat consumption. In addition to our initial pilot study ^20^, we have further piloted the questionnaires and scripts in each center among both men and women, both with different educational backgrounds, and of different ages to ensure readability and understandability in the general population. We have trained research staff and we will monitor study procedures to ensure quality implementation throughout the interview process. Ultimately, we will follow-up participants to determine if they have made any changes in their meat consumption according to what they have reported during the initial interview; this will allow us to assess the consistency and reliability of our study findings.

### Implications for practice and research

Our international study has direct implications for decision makers, guideline developers and policy makers in the development of nutritional recommendations. Up to now, this aspect has been neglected when formulating recommendations. Panels will now have access to international research evidence on values and preferences specific to actual estimated risk reductions in cancer, and the relevant certainty, associated with decreased meat intake. Based on international GRADE standards ^41^, this information will prove crucial for guideline panels moving from the evidence to recommendations on red and processed meat.

One potential area of further research will be the evaluation of how panels are using this new evidence when formulating recommendations. This work will also inform clinicians regarding community values and preferences when considering the implementation of diet related changes with their patients. Our proposal will use innovative approaches to assess people’s health values and preferences in relation to their diet. The study will provide a rigorous and transparent methodology that can be further utilized in the context of other nutritional scenarios.

## Data availability

### Underlying data

No data are associated with the article.

### Extended data

- Open Science Framework: https://doi.org/10.17605/OSF.IO/4HKXQ ^32^.
  - All servings/week scenarios for processed meat and for unprocessed red meat intake,
  - Semi-structured interview script
  - Follow-up assessment script.
- Spanish version of the online survey available here.

Data are available under the terms of the Creative Commons Attribution 4.0 International license (CC-BY 4.0).
