# Supplementary material for: Evaluating adults’ health-related values and preferences about unprocessed red meat and processed meat consumption: protocol for a cross-sectional mixed-methods study
Source: F1000Res. 2021 May 13;9:346. Originally published 2020 May 11. [Version 2] doi: 10.12688/f1000research.23593.2 (PMC8176263; doi:10.12688/f1000research.23593.2)
Supplement: Peer review history

# Peer review: Reviewer response for version 2

**DOI:** 10.5256/f1000research.56727.r85342

**Published:** 2021-06-03

**Authors:** Tomás Meroño

**Affiliations:** 1 Biomarkers and Nutrimetabolomics Laboratory, Department of Nutrition, Food Sciences and Gastronomy, Food Technology Reference Net (XaRTA), Nutrition and Food Safety Research Institute (INSA), Faculty of Pharmacy and Food Sciences, University of Barcelona, Barcelona, Spain

The authors improved the manuscript and answered all the points raised.

Is the study design appropriate for the research question?

Yes

Is the rationale for, and objectives of, the study clearly described?

Yes

Are sufficient details of the methods provided to allow replication by others?

Partly

Are the datasets clearly presented in a useable and accessible format?

Yes

Reviewer Expertise:

Nutrition, nutritional epidemiology.

I confirm that I have read this submission and believe that I have an appropriate level of expertise to confirm that it is of an acceptable scientific standard.

# Peer review: Reviewer response for version 2

**DOI:** 10.5256/f1000research.56727.r85341

**Published:** 2021-05-19

**Authors:** Kevin Maki, Mary Dicklin

**Affiliations:** 1 Midwest Biomedical Research, Addison, IL, USA; 2 Department of Applied Health Science, School of Public Health, Indiana University, Bloomington, IN, USA

This paper has been revised to address the comments from the reviewers, and I have just a few, relatively minor, suggestions for revisions as described below.

1. It might be useful to define the range of the Likert Scale (e.g., a 7-point scale from 1 [meaning definitely not] to 7 [meaning definitely yes]) where it is 1 ^st^ mentioned (i.e., in the Sample size section). It currently isn’t defined until later in the Direct choice exercise section, but it is mentioned earlier in both the Sample size and Study procedures sections.
2. In the 3 ^rd^ paragraph of the Study procedures section: In the sentence beginning, “If participants declared...” the word “unprocessed” is missing where it says the participants “will skip the questions on red meat”.
3. There appear to be some errors in the Semi-structured interview script available online: a) “unprocessed red meant” should be “unprocessed red meat”; b) perhaps “In which way” should be “In which ways do”; c) should the question that begins with “Are these factors...” refer to both processed meat and unprocessed red meat? d) “What could motive” should be “What could motivate”; e) The last question, “Do you think it is something that we would be interested in learning from the interviewees?” is unclear. Should this instead say something like “Is there anything that you think we would be interested in learning from the interviewees?”

Is the study design appropriate for the research question?

Partly

Is the rationale for, and objectives of, the study clearly described?

Yes

Are sufficient details of the methods provided to allow replication by others?

Partly

Are the datasets clearly presented in a useable and accessible format?

Not applicable

Reviewer Expertise:

Design and conduct of clinical studies in human nutrition, metabolism and chronic disease risk factor management.

We confirm that we have read this submission and believe that we have an appropriate level of expertise to confirm that it is of an acceptable scientific standard.

# Peer review: Reviewer response for version 1

**DOI:** 10.5256/f1000research.26034.r79343

**Published:** 2021-02-23

**Authors:** Tomás Meroño

**Affiliations:** 1 Biomarkers and Nutrimetabolomics Laboratory, Department of Nutrition, Food Sciences and Gastronomy, Food Technology Reference Net (XaRTA), Nutrition and Food Safety Research Institute (INSA), Faculty of Pharmacy and Food Sciences, University of Barcelona, Barcelona, Spain

The study protocol presented by Valli *et al.* aims to evaluate the willingness to change their consumption of unprocessed and processed red meat in light of risk reductions in cancer incidence and mortality. The "mixed-methods" refers to the combination of quantitative and qualitative research methodologies. The qualitative research aims to study the factors behind the willingness or not to modify red meat consumption.

 In addition, they propose to evaluate the preferences of consumption of the participants; however the relevance for this data is not clear in the actual version of the protocol.

**General comments**

- From a participant point of view, the tables with information of cancer incidence and mortality are more shocking than the low certainty estimates produced by the MAGICApp. Also, the figure is not of easy understanding. It does not translate in a clear message of what will happen to me if I do not reduce my red meat consumption.
- The meta-analysis from which estimates of risk reduction are taken has low to very low certainty in every case. Maybe the rather low level of certainty undermines the risk estimate impact on changing the consumption of red meat. What is the rationale behind choosing cancer despite of the low certainty evidence supporting the meta-analysis?
- I don't see clearly what is the rationale behind the assessments of the preferences on red meat consumption if the red meat target is the same for all the populations. How is this information going to be used in the analysis? Why is it important?
- The sample size of the subsample for the qualitative study is not stated. Moreover, which are the domains or the main issues in which the participants will be interviewed? If it is semi-structured, then the manuscript will require a brief description of the main questions or topics.
- I am wondering which would constitute a better outcome: i) the willingness to reduce red meat consumption or ii) the reduction of red meat consumption in the follow-up survey. In my opinion, the intervention is the initial survey and the result is the reduction of red meat consumption. Actually, we do not expect changes or at least there is not a pre and post examination after the direct-choice exercise.
- Does risk reductions from the meta-analysis consider differences in age, sex and smoking? 
- Last, how the research team will address the differences between country, site and type of interview when doing the statistical analysis. My guess is that participants in face-to-face interviews will feel pushed towards a "positive" answer, while the ones taking the online survey might be free of this effect. Perhaps, a subsample of the participants could take both surveys to see if such an effect is present and the evaluations are consistent one with the other.

**Minor comments** 

- Reference number 5 is not adequate for supporting the claim in the text. Please use a more relevant one.
- Page 3, Study population. When describing the exclusion criteria please delete the word "severe" cardiovascular disease.
- Sample size, instead of +/- 10, 0.1.
- What is the rationale for recruiting 96 in each site? From sample size calculation I would say that 96 participants in total are enough. Will the results be analysed by site or all-together? Anyway, I think the researchers choice is valid enough but some explanation would be appreciated. The definition of the main outcome and its rather high probability to occur with a quite large confidence interval might be behind this somehow small sample size for a multicenter study. On the other hand, please manifest at least some approximation to the total number of interviews for the qualitative analysis.
- Page 6, some typos, please revise.
- Revise definition of outcomes.

Is the study design appropriate for the research question?

Yes

Is the rationale for, and objectives of, the study clearly described?

Yes

Are sufficient details of the methods provided to allow replication by others?

Partly

Are the datasets clearly presented in a useable and accessible format?

Yes

Reviewer Expertise:

Nutrition, nutritional epidemiology.

I confirm that I have read this submission and believe that I have an appropriate level of expertise to confirm that it is of an acceptable scientific standard, however I have significant reservations, as outlined above.

# Response

**Published:** 2021-05-06

**Authors:** Claudia Valli

**Affiliations:** Hospital Sant Pau and Institute Rec, Spain

**Comment:**

 The study protocol presented by Valli et al. aims to evaluate the willingness to change their consumption of unprocessed and processed red meat in light of risk reductions in cancer incidence and mortality. The "mixed-methods" refers to the combination of quantitative and qualitative research methodologies. The qualitative research aims to study the factors behind the willingness or not to modify red meat consumption. In addition, they propose to evaluate the preferences of consumption of the participants; however, the relevance for this data is not clear in the actual version of the protocol.

Reply:

 Participants’ values and preferences regarding meat intake will inform us on their related motives around their willingness or unwillingness to make any changes. We have improved the section of Outcomes in the protocol to better explain the relevance of such data.

**Comment:**

 From a participant point of view, the tables with information of cancer incidence and mortality are more shocking than the low certainty estimates produced by the MAGICApp. Also, the figure is not of easy understanding. It does not translate in a clear message of what will happen to me if I do not reduce my red meat consumption.

Reply:

 In the online survey, we will provide an explanatory video that will describe to participants how to read/interpret the data presented in the scenarios to facilitate their understanding. In addition, the online survey will provide an explicit text tailored to each scenario right below the image of the scenario. Below, we have provided the examples used with participants:

*According to a recent high quality systematic review of the available scientific literature, for people like you who consume 3 servings a week of processed meat, the probability of developing cancer is 18.5%. This means that 185 people out of 1,000 may develop cancer and 815 may not.*

*For people who consume 3 fewer servings per week of processed meat, the probability of developing cancer is 18.3%. This means that 183 people out of 1,000 may develop cancer and 817 may not. Overall, in a population of 1,000 people, 2 fewer people may develop cancer by consuming 3 fewer servings per week of processed meat compared to a population of 1,000 people who do not reduce their consumption.*

*In other words, a reduction of 3 servings per week of processed meat may reduce the probability of developing cancer and the certainty of this effect is very low. This means that the results we present come from studies with important limitations. Therefore, there is a possibility that a reduction in the consumption of processed meat may not result in a reduction in the risk of developing cancer.*

**Comment:**

 The meta-analysis from which estimates of risk reduction are taken has low to very low certainty in every case. Maybe the rather low level of certainty undermines the risk estimate impact on changing the consumption of red meat. What is the rationale behind choosing cancer despite of the low certainty evidence supporting the meta-analysis?

Reply:

 We have decided to prioritize cancer incidence and cancer mortality because we believed these two general outcomes would be very familiar and of general interest to the public. Regarding the certainty of the evidence, for all outcomes the certainty was low- to very low because of observational design as well as issues with imprecision and/or risk of bias (Han 2019). Among the cancer outcomes, we have selected those that demonstrated the largest risk reduction effect.  The certainty would be no higher had we selected cardiovascular outcomes, the other reasonable choice in terms of outcomes of most important to patients.

**Comment:**

 I don't see clearly what is the rationale behind the assessments of the preferences on red meat consumption if the red meat target is the same for all the populations. How is this information going to be used in the analysis? Why is it important?

Reply:

 Assessing the values and preferences from representative members of the population will inform us on the variability between the participants and between the countries, the objective of the study. We tailored our questions to each participant in each country depending on that average weekly intake. For instance, if participants consumed 6 servings of unprocessed red meat per week, we showed them the absolute risk reduction if they were to consume 6 fewer servings per week (stop completely), as well as 3 fewer servings in case they were unwilling to stop completely. Values and preferences from those who will actually use guideline recommendations, in this case -- members of the public -- are an important component of guideline development (Dedios 2017; Erickson 2017).

**Comment:**

 The sample size of the subsample for the qualitative study is not stated. Moreover, which are the domains or the main issues in which the participants will be interviewed? If it is semi-structured, then the manuscript will require a brief description of the main questions or topics.

Reply:

 For qualitative evaluations, it is unusual to predetermine the sample size. As stated, and referenced in the manuscript, the number of needed participants will be determined by data saturation method, that is, we will continue interviewing new participants until no additional concepts or themes emerge.

 For the semi-structured interview, we have provided the draft of questionnaire as a supplementary material of the protocol. Please note, that being a semi-structured interview, questions might change and be adapted according to the conversation with the participant and to his/her responses given in the online survey.

**Comment:**

 I am wondering which would constitute a better outcome: i) the willingness to reduce red meat consumption or ii) the reduction of red meat consumption in the follow-up survey. In my opinion, the intervention is the initial survey and the result is the reduction of red meat consumption. Actually, we do not expect changes or at least there is not a pre- and post-examination after the direct-choice exercise.

Reply:

 Not totally clear to us what the reviewer means by “better”. From some peoples’ perspective a good outcome might be that respondents who say they are willing to reduce their meat consumption as a result of the information presented would actually do so. Our aim is to assess both outcomes, report the results and reflect on their implications.

 To clarify, the outcome “willingness” will inform us about participants willingness to change only after being informed on the potential undesirable health outcomes, the first study of its kind to our knowledge. On the other hand, assessing participants’ meat consumption in the follow-up will show us if participants have made any changes in their meat intake compared to what they reported to consume in the online survey and also compared to what they affirmed to be willing to do in the direct-choice exercise (3 months earlier). This will allow us to detect if participants have been made any changes.

**Comment:**

 Do risk reductions from the meta-analysis consider differences in age, sex and smoking?

Reply:

 Some of the studies included in the meta-analysis had limitations due to limited adjustment for potential confounders (Han 2019). That means that some studies took into account potential confounders such as age and sex and smoking but others did not. Lack of adequate adjustment for potential confounders (e.g., age, sex, smoking, socioeconomic status, exercise etc.) was one of the main sources of potential bias. Limitations in adjustment was an important reason for rating down the certainty of the evidence.

**Comment:**

 Last, how the research team will address the differences between country, site and type of interview when doing the statistical analysis. My guess is that participants in face-to-face interviews will feel pushed towards a "positive" answer, while the ones taking the online survey might be free of this effect. Perhaps, a subsample of the participants could take both surveys to see if such an effect is present and the evaluations are consistent one with the other.

Reply:

 Thanks for the suggestion to compare responses online versus face-to-face surveys. Due to the COVID-19 pandemic, all sites will conduct the study remotely through an online survey. The pilot study was conducted in-person since it was implemented before the start of the pandemic. Each country will run their own analysis separately resulting in four different publications.

**Comment:**

 Reference number 5 is not adequate for supporting the claim in the text. Please use a more relevant one.

Reply:

 We believe the said reference *Rabassa* *2018* is appropriate since it refers to a recent study assessing the quality of nutrition guidelines with the use of the AGREE tool, an internationally recognized instrument used to evaluate the process of practice guideline development and the quality of reporting. We have added an additional reference *Blake 2018*, which is an analysis the appropriateness of methods used to synthesize evidence and grade recommendations in food-based dietary guidelines.

**Comment:**

 Page 3, Study population. When describing the exclusion criteria please delete the word "severe" cardiovascular disease.

Reply:

 Thank you, we have edited the text according to the reviewer’s suggestion.

**Comment:**

 Sample size, instead of +/- 10, 0.1.

Reply:

 Thank you, we have edited the text by following the reviewers’ suggestions.

**Comment:**

 What is the rationale for recruiting 96 in each site? From sample size calculation I would say that 96 participants in total are enough. Will the results be analysed by site or all-together? Anyway, I think the researcher’s choice is valid enough but some explanation would be appreciated. The definition of the main outcome and its rather high probability to occur with a quite large confidence interval might be behind this somehow small sample size for a multicenter study. On the other hand, please manifest at least some approximation to the total number of interviews for the qualitative analysis.

Reply:

 The more variable the response, the larger the sample size necessary to assess whether observed results are representative of the target population or simply reflects random variation. The sample size calculation does not take into account the multi-centred nature of the study. Since we are conducting the study in different sites/countries and considering the cultural differences, the responses of approximately 20 participants in each site/country (96 participants in total) will not give us representative results within each country. Therefore, we have used a more conservative approach by attempting to recruit at least 96 participants per site. As stated above, for qualitative evaluations, it is unusual to predetermine the sample size, rather we will sample participants until we reach data saturation, meaning that we will continue interviewing new participants until no additional concepts or themes emerge.

**Comment:**

 Page 6, some typos, please revise.

 Revise definition of outcomes.

Reply:

 Thank you, we have made the corrections in the text.

 References

- World Cancer Research Fund, American Institute for Cancer Research. Food, nutrition, physical activity, and the prevention of cancer: a global perspective. Amer Inst for Cancer Research; 2007.
- Han MA, Zeraatkar D, Guyatt GH, Vernooij RWM, El Dib R, Zhang Y, Algarni A, Leung G, Storman D, Valli C, Rabassa M, Rehman N, Parvizian MK, Zworth M, Bartoszko JJ, Lopes LC, Sit D, Bala MM, Alonso-Coello P, Johnston BC. Reduction of Red and Processed Meat Intake and Cancer Mortality and Incidence: A Systematic Review and Meta-analysis of Cohort Studies. Ann Intern Med. 2019.
- Blake P, Durão S, Naude CE, Bero L. An analysis of methods used to synthesize evidence and grade recommendations in food-based dietary guidelines. Nutr Rev. 2018; 76(4):290-300.
- Rabassa M, Garcia-Ribera Ruiz S, Solà I, Pardo-Hernandez H, Alonso-Coello P, García LM. Nutrition Guidelines vary widely in methodological quality: An overview of reviews. J Clin Epidemiol. 2018; pii: S0895-4356(18)30374-3
- Thomson R. Decision analysis - utility for everyday use? Shared decision-making in health care: achieving evidence-based patient choice (paperback). Edited by: Edwards A, Elwyn G. 2009, Oxford University Press, Oxford
- Thomson R, Robinson A, Greenaway J, Lowe P: Development and description of a decision analysis-based decision support tool for stroke prevention in atrial fibrillation. Qual Saf Health Care. 2002, 11: 25-10.
- Erickson J, Sadeghirad B, Lytvyn L, Slavin J, Johnston BC; The Scientific Basis of Guideline Recommendations on Sugar Intake: A Systematic Review. Ann Intern Med. 2017;166(4):257-267.
- Dedios MC ,  Esperato A ,  De-Regil LM , et al. Improving the adaptability of WHO evidence-informed guidelines for nutrition actions: results of a mixed methods evaluation. Implement Sci. 2017;12:39. 

# Peer review: Reviewer response for version 1

**DOI:** 10.5256/f1000research.26034.r72360

**Published:** 2020-10-21

**Authors:** Kevin Maki, Mary Dicklin

**Affiliations:** 1 Midwest Biomedical Research, Addison, IL, USA; 2 Department of Applied Health Science, School of Public Health, Indiana University, Bloomington, IN, USA

This paper describes a protocol for a study designed to investigate the potential impact on participants’ thoughts and actions with regard to reducing or avoiding red and processed meat intake when educated about the potential effects on cancer incidence and cancer mortality. The study began in 2019 with expected completion early next year (2021).

1. The NutriRECS investigation has grouped unprocessed red meat and processed meat into a single category. It is not clear in the description of the questionnaires for this study whether these will be distinguished. Entry for the study is based on consumption of a minimum of 3 servings/week of either unprocessed red meat or processed meat. It appears that the investigators will only be asking the participants 1 question about their willingness to stop or reduce “consuming either unprocessed red meat or processed meat (whichever they are eating more of).” The “whichever they are eating more of” part of the question is confusing. I think a better approach would be to investigate their willingness to stop or reduce consumption of each of these types of meats in separate questions.
2. Does “four different sites in four countries” mean a total of 16 sites, which would be a total of 1536 participants, or 4 sites total, which would be 484 participants?
3. The participants’ educational backgrounds are being analyzed, but do your questionnaires also ask the participants about their baseline knowledge of the potential relation between diet and cancer (including specific types of cancer)?
4. In the sample size section, you refer to the proportions as 0.44 (44%) and 0.5 (50%), but then indicate that a confidence interval of ±10 is acceptable. I think this should be 0.1 for consistency.
5. Can you make the current meat consumption questionnaire available as supplemental material? Also, can you please provide more details about the question(s) that will be asked of the participants at the 3-month follow-up interview?
6. Can you provide data showing the average intake of unprocessed red meat vs. processed meat in Poland, instead of total unprocessed and processed meat that you have reported?
7. Table 1: In the 2 ^nd^ bullet point, I think “other” should be “others are”; something appears to be missing in “a combination of subsequent is needed.”
8. Table 2: Some of the statements about mortality seem obvious, and I am not sure how they will be interpreted by the participants, i.e., “You are dead and you do not feel any pain or breathlessness” and “You will leave everything that was important in short time span”.
9. Figures 1 and 2: Although these figures will likely be understandable if described to the participants by the investigator, as stand-alone figures, the “2 fewer” and “9 fewer” may not be clear to them. I think the statement “Among a 1000 patients like you…” could be expanded to include a description of the 2 fewer (or 9 fewer) to make this clearer.
10. In a few places you refer to a “subsample” but it is not clear in the Study procedures section where you discuss asking additional questions about motives that this is the subsample.
11. Will you analyze the data separately according to countries/sites?
12. In the Quantitative analysis section you say that you will describe “willingness to reduce meat consumption in the face of undesirable cancer health risks” and “willingness to avoid meat consumption in the face of the undesirable cancer health risks.” I think these should specify that the meat is red and processed meats.
13. I appreciate your desire to “not overburden participants with too much information, we prioritized two cancer outcomes” – cancer incidence and cancer mortality. However, I wonder in your interviews with the participants if you will be providing them with more information about the relation between meat intake and specific types of cancers, e.g., colorectal, breast, etc.

Is the study design appropriate for the research question?

Partly

Is the rationale for, and objectives of, the study clearly described?

Yes

Are sufficient details of the methods provided to allow replication by others?

Partly

Are the datasets clearly presented in a useable and accessible format?

Not applicable

Reviewer Expertise:

Design and conduct of clinical studies in human nutrition, metabolism, and chronic disease risk factor management.

We confirm that we have read this submission and believe that we have an appropriate level of expertise to confirm that it is of an acceptable scientific standard, however we have significant reservations, as outlined above.

# Response

**Published:** 2021-05-06

**Authors:** Claudia Valli

**Affiliations:** Hospital Sant Pau and Institute Rec, Spain

**Comment:**

 This paper describes a protocol for a study designed to investigate the potential impact on participants’ thoughts and actions with regard to reducing or avoiding red and processed meat intake when educated about the potential effects on cancer incidence and cancer mortality. The study began in 2019 with expected completion early next year (2021).

 The NutriRECS investigation has grouped unprocessed red meat and processed meat into a single category. It is not clear in the description of the questionnaires for this study whether these will be distinguished. Entry for the study is based on consumption of a minimum of 3 servings/week of either unprocessed red meat or processed meat. It appears that the investigators will only be asking the participants 1 question about their willingness to stop or reduce “consuming either unprocessed red meat or processed meat (whichever they are eating more of).” The “whichever they are eating more of” part of the question is confusing. I think a better approach would be to investigate their willingness to stop or reduce consumption of each of these types of meats in separate questions.

Reply:

 We would like to clarify that red meat and processed meat have not been considered as one category, instead they will be presented to participants as two different types of meat. Unprocessed red meat is defined as mammalian meat (e.g., beef, pork, lamb) and processed meat is defined as white or red meat preserved by smoking, curing, salting, or by the addition of preservatives (e.g., hot dogs, charcuterie, sausage, ham, and cold cut deli meats) (World Cancer Research Fund, 2007).

 We will include participants consuming a minimum of 3 servings per week of either unprocessed red meat or processed meat. Each participant will be presented with one question to assess their unprocessed red meat intake and one question to assess their processed meat intake per week. That means we will determine unprocessed red meat intake and processed meat intake separately for all included participants. Based on the reported intake of unprocessed red meat and processed meat, participants will be presented with scenarios tailored to their consumption. After presenting them with the scenario, participants will be asked series of questions regarding their willingness to change their meat intake. First, participants will be presented with one question about their willingness to stop eating unprocessed red meat. If participants are unwilling to stop (≤4 of the Likert-scale), they will be presented with an additional question about their willingness to reduce. In case participants are willing to stop their unprocessed red meat intake (≥5 of the Likert-scale), the questions on reduction will be no longer relevant. Subsequently, for those that consume processed meat, they will be presented with the scenario on processed red meat and they will be asked to answer a question about their willingness to stop eating processed meat. If participants are willing to stop processed meat, we will use the same logic for questions explained above.

 That means that we will determine participants’ willingness to stop eating unprocessed red meat and participants’ willingness to stop eating processed meat separately. Additionally, for those participants who report being unwilling to stop, we will determine their willingness to reduce unprocessed meat and their willingness to reduce processed meat intake.

**Comment:**

 Does “four different sites in four countries” mean a total of 16 sites, which would be a total of 1536 participants, or 4 sites total, which would be 484 participants?

Reply:

 To clarify, the study is conducted in 3 different sites in total (one site in each country: Spain, Poland and Brazil). In 2019, we conducted a pilot study in Canada allowing us to obtain preliminary data to inform our sample size calculation. Based on the pilot data, we calculated a sample size of at least 96 participants per site.

**Comment:**

 The participants’ educational backgrounds are being analysed, but do your questionnaires also ask the participants about their baseline knowledge of the potential relation between diet and cancer (including specific types of cancer)?

Reply:

 Although we inquired their consumption of meat related to health, we did not specifically ask participants questions about their knowledge regarding potential cancer risks associated with dietary behaviours. Instead, we asked participants if they had reduced their unprocessed red meat and processed meat intake in the past because of health reasons.

**Comment:**

 In the sample size section, you refer to the proportions as 0.44 (44%) and 0.5 (50%), but then indicate that a confidence interval of ±10 is acceptable. I think this should be 0.1 for consistency.

Reply:

 Thank you, we have edited the text to correct this oversight.

**Comment:**

 Can you make the current meat consumption questionnaire available as supplemental material? Also, can you please provide more details about the question(s) that will be asked of the participants at the 3-month follow-up interview?

Reply:

 Due to the COVID-19 pandemic, we administered the questionnaire online and thus, we do not have the final version on paper in English. We have provided access through a link in the protocol to the Spanish version of the online survey. The only survey in English available is the one used in the pilot study in Canada and therefore, does not represent the final version used. For the 3- months follow up assessment we have provided the questionnaire as a supplementary material of the protocol.

**Comment:**

 Can you provide data showing the average intake of unprocessed red meat vs. processed meat in Poland, instead of total unprocessed and processed meat that you have reported?

Reply:

 The information on meat consumption in Poland was based on the data available at Statistics Poland https://stat.gov.pl/en/topics/prices-trade/trade/domestic-deliveries-and-consumption-of-selected-consumer-goods-per-capita-in-2018,9,8.html, which is the main Polish agency offering a national-wide data. Unfortunately, the available data did not report unprocessed red meat and processed meat separately.

**Comment:**

 Table 1: In the 2 ^nd^ bullet point, I think “other” should be “others are”; something appears to be missing in “a combination of subsequent is needed.”

Reply:

 Thank you, we have corrected Table 1 following the reviewers’ suggestions.

**Comment:**

 Table 2: Some of the statements about mortality seem obvious, and I am not sure how they will be interpreted by the participants, i.e., “You are dead and you do not feel any pain or breathlessness” and “You will leave everything that was important in short time span”.

Reply:

 The application of health states is commonly used in studies that elicit participants’ health values and preference to help ensure a similar understanding of the presented health outcomes (Thomson 2002; Thomson 2009). Although, the cancer mortality health state seems to be obvious, the symptoms, treatments and consequences of dying of cancer are not the same as the ones for dying of other diseases, for example heart failure. Thus, we believe it is helpful to provide the descriptions of both outcomes separately.

**Comment:**

 Figures 1 and 2: Although these figures will likely be understandable if described to the participants by the investigator, as stand-alone figures, the “2 fewer” and “9 fewer” may not be clear to them. I think the statement “Among 1000 patients like you…” could be expanded to include a description of the 2 fewer (or 9 fewer) to make this clearer.

Reply:

 In the online survey, we are providing an explanatory video that will describe to participants how to read/interpret the data presented in the scenarios to facilitate their understanding. In addition, we will provide an explicit text tailored to each scenario. Below, we have provided examples:

*According to a recent high quality systematic review of the available scientific literature, for people like you who consume 3 servings a week of processed meat, the probability of developing cancer is 18.5%. This means that 185 people out of 1,000 may develop cancer and 815 may not.*

*For people who consume 3 fewer servings per week of processed meat, the probability of developing cancer is 18.3%. This means that 183 people out of 1,000 may develop cancer and 817 may not. Overall, in a population of 1,000 people, 2 fewer people may develop cancer by consuming 3 fewer servings per week of processed meat compared to a population of 1,000 people who do not reduce their consumption.*

*In other words, a reduction of 3 servings per week of processed meat may reduce the probability of developing cancer and the certainty of this effect is very low. This means that the results we present come from studies with important limitations. Therefore, there is a possibility that a reduction in the consumption of processed meat may not result in a reduction in the risk of developing cancer.*

**Comment:**

 In a few places you refer to a “subsample” but it is not clear in the Study procedures section where you discuss asking additional questions about motives that this is the subsample.

Reply:

 We have addressed the reviewers’ comments in the protocol and removed the term subsample to avoid confusion.

**Comment:**

 Will you analyze the data separately according to countries/sites?

Reply:

 Each country will run their own analysis separately that will result in three different publications. Additionally, the pilot study methods and results will also be published in a separate publication.

**Comment:**

 In the Quantitative analysis section, you say that you will describe “willingness to reduce meat consumption in the face of undesirable cancer health risks” and “willingness to avoid meat consumption in the face of the undesirable cancer health risks.” I think these should specify that the meat is red and processed meats.

Reply:

 We have addressed and have now added “red and processed” meat in the protocol.

**Comment:**

 I appreciate your desire to “not overburden participants with too much information, we prioritized two cancer outcomes” – cancer incidence and cancer mortality. However, I wonder in your interviews with the participants if you will be providing them with more information about the relation between meat intake and specific types of cancers, e.g., colorectal, breast, etc.

Reply:

 We have decided to prioritize cancer incidence and cancer mortality because we believed these two general outcomes would be more familiar to the general population. Also, cancer mortality and cancer incidence were the two outcomes showing the largest absolute risk reduction. Being a cross-sectional study requiring participant’s involvement, we had to consider the length of the survey and time people would be willing to dedicate to the study.

 References

- World Cancer Research Fund, American Institute for Cancer Research. Food, nutrition, physical activity, and the prevention of cancer: a global perspective. Amer Inst for Cancer Research; 2007.
- Han MA, Zeraatkar D, Guyatt GH, Vernooij RWM, El Dib R, Zhang Y, Algarni A, Leung G, Storman D, Valli C, Rabassa M, Rehman N, Parvizian MK, Zworth M, Bartoszko JJ, Lopes LC, Sit D, Bala MM, Alonso-Coello P, Johnston BC. Reduction of Red and Processed Meat Intake and Cancer Mortality and Incidence: A Systematic Review and Meta-analysis of Cohort Studies. Ann Intern Med. 2019.
- Blake P, Durão S, Naude CE, Bero L. An analysis of methods used to synthesize evidence and grade recommendations in food-based dietary guidelines. Nutr Rev. 2018; 76(4):290-300.
- Rabassa M, Garcia-Ribera Ruiz S, Solà I, Pardo-Hernandez H, Alonso-Coello P, García LM. Nutrition Guidelines vary widely in methodological quality: An overview of reviews. J Clin Epidemiol. 2018; pii: S0895-4356(18)30374-3
- Thomson R. Decision analysis - utility for everyday use? Shared decision-making in health care: achieving evidence-based patient choice (paperback). Edited by: Edwards A, Elwyn G. 2009, Oxford University Press, Oxford
- Thomson R, Robinson A, Greenaway J, Lowe P: Development and description of a decision analysis-based decision support tool for stroke prevention in atrial fibrillation. Qual Saf Health Care. 2002, 11: 25-10.
- Erickson J, Sadeghirad B, Lytvyn L, Slavin J, Johnston BC; The Scientific Basis of Guideline Recommendations on Sugar Intake: A Systematic Review. Ann Intern Med. 2017;166(4):257-267.
- Dedios MC ,  Esperato A ,  De-Regil LM , et al. Improving the adaptability of WHO evidence-informed guidelines for nutrition actions: results of a mixed methods evaluation. Implement Sci. 2017;12:39. 
